# Gambogic acid: Multi-gram scale isolation, stereochemical erosion toward epi-gambogic acid and biological profile

**DOI:** 10.3389/fntpr.2022.1018765

**Published:** 2022

**Authors:** Gary E. Arevalo, Michelle K. Frank, Katelin S. Decker, Maria A. Theodoraki, Emmanuel A. Theodorakis

**Affiliations:** 1 Department of Chemistry and Biochemistry, University of California, San Diego, La Jolla, CA, United States; 2 Department of Biology, Arcadia University, Glenside, PA, United States

**Keywords:** natural product, synthetic methods, garcinia, gamboge, breast cancer

## Abstract

**Introduction:**

Extracted from gamboge resin, gambogic acid (**GBA**) is a natural product that displays a complex caged xanthone structure and exhibits promising antitumor properties. However, efforts to advance this compound to clinical applications have been thwarted by its limited availability that in turn, restricts its pharmacological optimization.

**Methods:**

We report here an efficient method that allows multigram scale isolation of **GBA** in greater than 97% diastereomeric purity from various sources of commercially available gamboge. The overall process includes: (a) isolation of organic components from the resin; (b) separation of **GBA** from the organic components via crystallization as its pyridinium salt; and (c) acidification of the salt to isolate the free **GBA**.

**Results and Discussion:**

We found that **GBA** is susceptible to epimerization at the C2 center that produces *epi*-gambogic acid (***epi*-GBA**), a common contaminant of all commercial sources of this compound. Mechanistic studies indicate that this epimerization proceeds *via* an *ortho*-quinone methide intermediate. Although the observed stereochemical erosion accounts for the chemical fragility of **GBA**, it does not significantly affect its biological activity especially as it relates to cancer cell cytotoxicity. Specifically, we measured similar levels of cytotoxicity for either pure **GBA** or an equilibrated mixture of **GBA**/ ***epi*-GBA** in MBA-MB-231 cells with IC_50_ values at submicromolar concentration and induction of apoptosis after 12 hours of incubation. The results validate the pharmacological promise of gambogic acid and, combined with the multigram-scale isolation, should enable drug design and development studies.

## Graphical Abstract

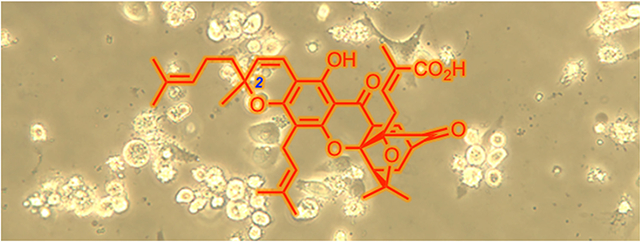

## 1 Introduction

**FIGURE 1:**
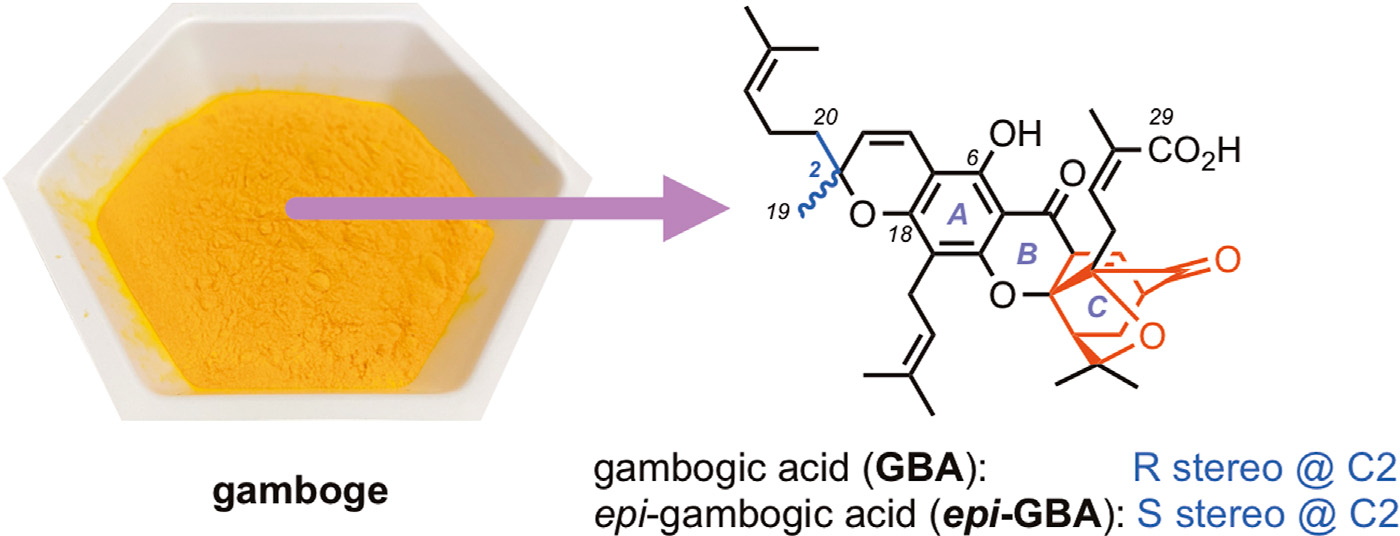
Chemical structures of gambogic acid (**GBA**) and its C2 epimer *epi*-gambogic acid (***epi*-GBA**) available from natural gamboge (yellow powder). The cage motif of these compounds and related **CGX**s is shown in red.

Tropical trees of the genus *Garcinia*, grown mainly in Southeast Asia, Brazil and India, are widely known not only for their high value as sources of food but also for their impact in arts and sciences (Sweeney, 2008; Hemshekhar et al., 2011; Kumar et al., 2013; Maneenoon et al., 2015; Espirito Santo et al., 2020). For instance gamboge, the yellow resin from *Garcinia* spp., has been used as a colorant for various artifacts and paintings around the world (Figure 1). (Fitzhugh, 1997; Eremin et al., 2006) Also known as “rongthong” (gold resin) gamboge has been used in Eastern ethnomedicine for its anti-infective and anti-parasitic properties (Panthong et al., 2007; Wang et al., 2018; Yu et al., 2022). Efforts to isolate the bioactive constituents of gamboge led to the identification of gambogic acid (**GBA**, Figure 1), an unusual polyprenylated metabolite structurally defined by a tricyclic xanthone backbone (ABC ring system) of which the C-ring has been converted into a cage structure (Yates et al., 1963; Ren et al., 2011; Xu et al., 2017). The seemingly inconspicuous xanthone motif is further decorated by peripheral substitutions and oxidations to produce an ever-growing family of natural products collectively referred to as caged *Garcinia* xanthones (**CGXs**) (Chantarasriwong et al., 2010; Anantachoke et al., 2012; Jia et al., 2015; Chantarasriwong et al., 2018; Phang et al., 2022).

Aside from their striking architecture, most caged xanthone family members display promising biological activities and are considered attractive candidates for drug design. For instance, **GBA** exhibits a potent antitumor profile as evidenced by its ability to inhibit cancer cell growth, invasion, metastasis and angiogenesis in various cell-based assays (Wang and Chen, 2012; Hatami et al., 2020; Liu et al., 2020; Li et al., 2022). Although the exact biological mode of action of this natural product is still under study, **GBA** has emerged as a potent inhibitor of heat-shock protein 90 (HSP90) (Zhang et al., 2010; Davenport et al., 2011; Elbel et al., 2013; Yim et al., 2016). Other recent proteomic studies have shown that this natural product targets or affects the expression of several proteins that are involved in cancer growth and development (Wang et al., 2009; Li et al., 2015; Hoch et al., 2020; Xia and Tang, 2021). Moreover, pharmacological and toxicity studies in animal models have shown that **GBA** has minimal effects on cardiovascular and respiratory functions suggesting a favorable safety profile and acceptable therapeutic index (Guo et al., 2006; Qi et al., 2008; Zhao et al., 2010; Yang et al., 2011). In fact, **GBA** has entered clinical trials in China for the treatment of non-small cell lung, colon and renal cancers (Chi et al., 2013). Albeit promising, these studies have identified certain challenges in developing **GBA** as a drug that may stem from its limited stability and suboptimal pharmacokinetics (Hatami et al., 2020; Liu et al., 2020). In principle, these challenges can be overcome by evaluating synthetic analogs, conjugates, and delivery systems for **GBA**. However, these studies are hindered by the limited availability of this natural product.

At present, gambogic acid is obtained from gamboge *via* extraction as its pyridinium salt followed by acidification (Weakley et al., 2001). This approach is not streamlined and results in **GBA** that is expensive and also available in milligram amounts. More importantly, commercially available **GBA** is typically contaminated with various amounts of its C2 epimer, a compound known as *epi-*gambogic acid (***epi*-GBA**, Figure 1). (Han et al., 2006a; Han et al., 2006b) In continuation of our studies on the chemistry and biology of **CGXs**, (Tisdale et al., 2004; Batova et al., 2007; Chantarasriwong et al., 2009; Batova et al., 2010; Yim et al., 2016; Ke et al., 2017), we present an efficient method to isolate **GBA** from readily available gamboge as a single isomer in greater than 97% purity. We also show that **GBA** can undergo a thermal isomerization to ***epi-*GBA** *via* a process that involves formation of an *ortho*-quinone methide intermediate and results in a nearly 6:4 ratio of the two compounds. This stereochemical erosion at the C2 stereocenter hints to inherent stability issues of this natural product. Nonetheless, we found that both **GBA** and ***epi*-GBA** show similar cytotoxicity effects in MDA-MB-231 breast cancer cells suggesting that the ubiquitous C2 isomerization does not significantly impact the bioactivity of this natural product.

## 2 Results and discussion

### 2.1 Isolation of gambogic acid from gamboge

**FIGURE 2:**
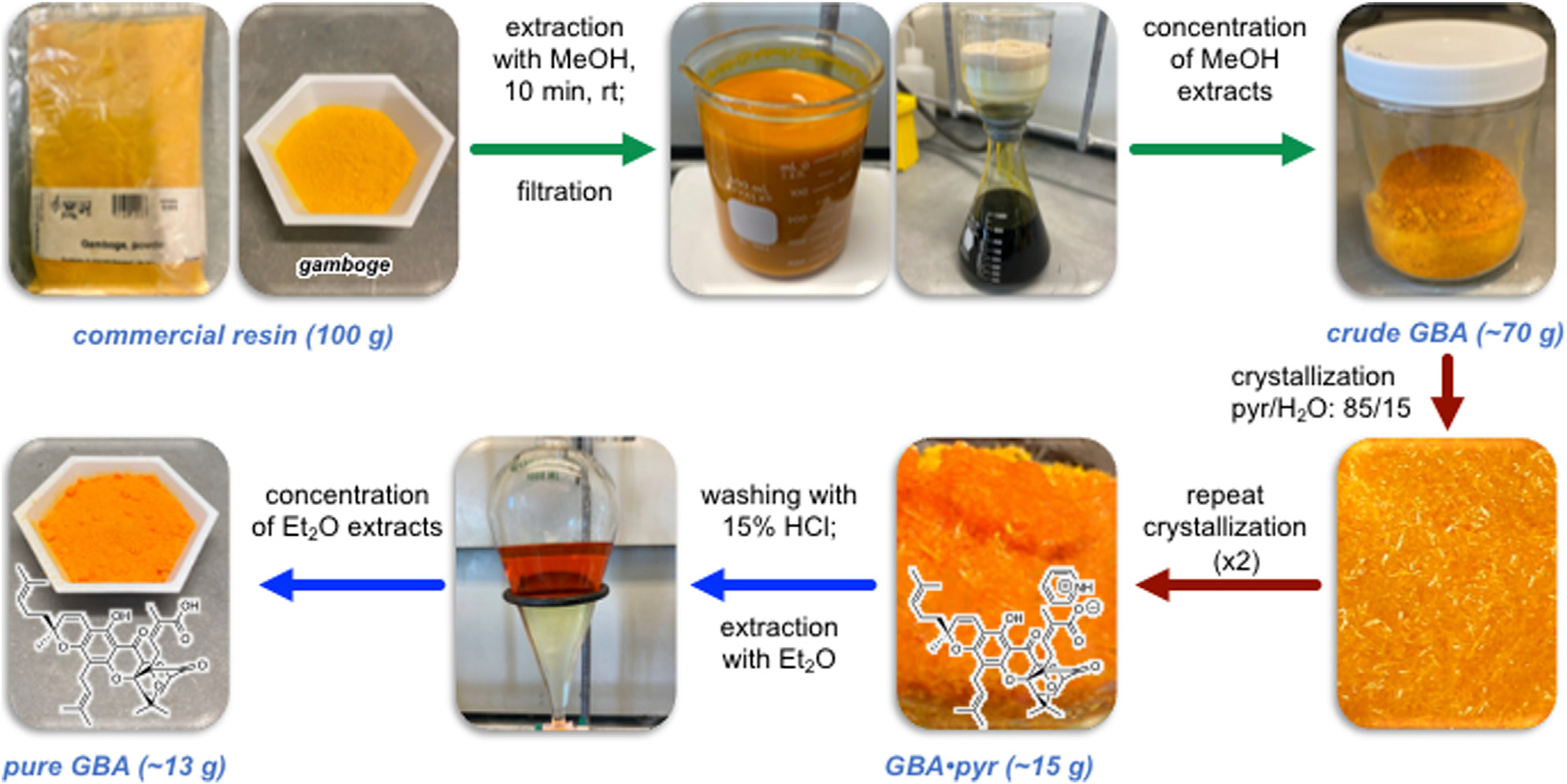
Workflow for the isolation of **GBA** from commercially available gamboge resin. Step 1 (green) involves extraction, filtration, and concentration of **crude GBA**. Step 2 (red) involves (re)crystallization and isolation of gambogic acid as its pyridinium salt (**GBA•pyr**). Step 3 (blue) involves neutralization of the pyridinium salt of gambogic acid followed by extraction/concentration to yield pure **GBA**.

The purification of **GBA** from commercially available gamboge was performed in three steps that include: 1) separation of components that are soluble in organic solvents from insoluble material; 2) separation of **GBA** from the organic components *via* crystallization as its pyridinium salt; and 3) isolation of free **GBA** (Figure 2). Previous studies have indicated that prolonged exposure of **GBA** to hydroxylated solvents gives rise to conjugate addition products (Han et al., 2005; Wang et al., 2010). With this in mind, we attempted to extract gamboge with acetone, acetonitrile, diethyl ether, and dichloromethane (Table S1, ESI). However, these solvents were not as efficient as methanol-based extraction and in certain cases led to swelling of the gamboge that, in turn, impeded filtration.

The most reproducible extraction protocol involves stirring of gamboge with methanol for 10 min followed by rapid filtration under reduced pressure and concentration to dryness. When performed on a 100 g scale, this process consistently leads to 70 g of a solid, referred to as **crude GBA** that is composed of approximately 30% **GBA**, 25% *epi-***GBA**, and 45% of other non-identified products as determined by ^1^H NMR spectroscopy (Supplementary Figure S1, ESI). Next, we focused on separating **GBA** from the above solid. We used pyridine as the extraction solvent since the pyridinium salt of gambogic acid (**GBA•pyr**) is known to be a crystalline solid. Although crystallization using pyridine as the only solvent was slow and inefficient, we found that adding water to the pyridine accelerated the crystallization of **GBA•pyr**. After experimenting with various ratios of water in pyridine, we found that an 85/15 mixture of pyridine/water leads to complete and rapid solubilization of **crude GBA** at 60°C and gives rise to high quality crystals of **GBA•pyr** after slow cooling to room temperature. Spectroscopic evaluation (^1^H NMR) showed that the resulting material was composed of 76% pyridinium salt of gambogic acid (**GBA•pyr**), 18% of pyridinium salt of *epi*-gambogic acid (***epi*-GBA•pyr**) and 6% of a non-identified compound (Supplementary Figure S2, ESI). A second crystallization under identical conditions led to a mixture of 89% **GBA•pyr**, 9% of ***epi*-GBA•pyr**, and 2% of a non-identified compound. A third crystallization afforded 14.9 g of orange crystals composed of 97% **GBA•pyr** and 3% ***epi*-GBA•pyr** (Supplementary Figures S3–S4, ESI). Subsequent crystallizations did not significantly improve the above ratio. The last step included treatment of **GBA•pyr** with 15% aqueous HCl and extraction of **GBA** with diethyl ether. This step generated pure **GBA** in quantitative yield while maintaining diastereomeric purity of >97% or a diastereomeric excess (d.e.) at C2 of >94% (Supplementary Figures S5–S6, ESI). It is worth noting that the C6 phenol of **GBA** resonates at 12.75 ppm in the ^1^H NMR spectrum in CDCl_3_ while that of ***epi*-GBA** resonates at 12.76 ppm facilitating d.e. measurements using ^1^H NMR spectroscopy.

To further evaluate the generality and reproducibility of the overall isolation workflow, we performed the above steps with gamboge resin purchased from three different suppliers (see Experimental Section). We found that the composition of organic extracts (i.e., **crude GBA**) was very similar in all cases and accounted for about 30% **GBA**, 25% ***epi*-GBA**, and 45% of other non-identifiable material (Supplementary Figure S7, ESI). Overall, starting from 100 g of any commercially available gamboge resin, this process can yield approximately 13 g of pure **GBA** in >97% purity (>94% d.e. at C2).

### 2.2 Isomerization of GBA to *epi*-GBA

**FIGURE 3:**
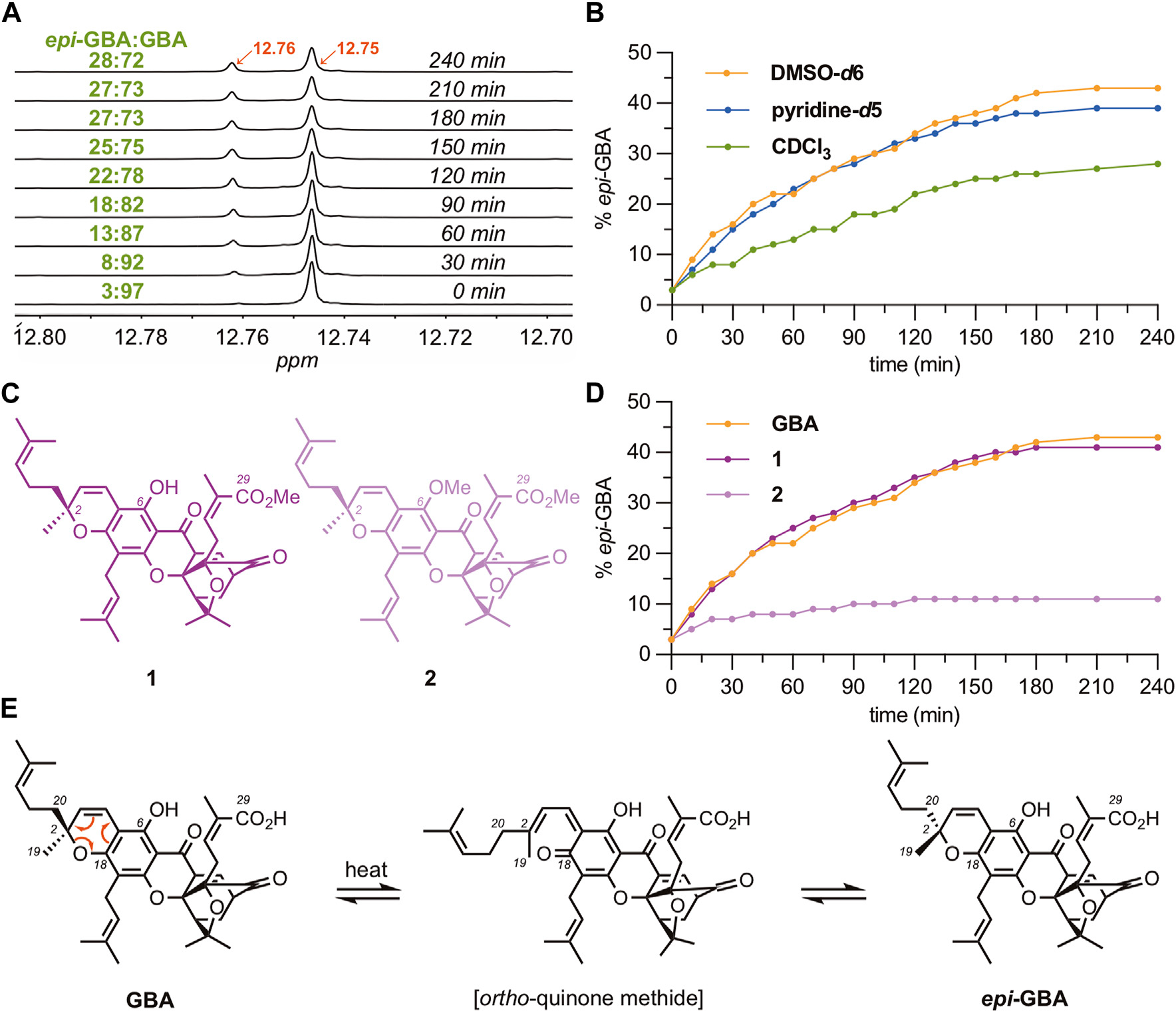
Epimerization studies of **GBA** to ***epi*-GBA**. **(A)** ^1^H NMR spectra showing formation of ***epi*-GBA** over time as a solution of pure **GBA** is heated in CDCl_3_ at 100°C (sealed NMR tube) over 4 h. **(B)** Graph of the rate of epimerization of **GBA** to ***epi*-GBA** in various solvents. **(C)** Chemical structure of compounds **1** and **2**, that possess one acidic proton and no acidic protons, respectively. **(D)** Rate of epimerization of **GBA**, **1**, and **2** in DMSO-*d*6 at 100°C over 4 h. **(E)** Proposed mechanism for the epimerization of **GBA** to ***epi*-GBA**.

Puzzled by the ubiquitous presence of ***epi*-GBA** in all steps and fractions discussed above, we tested if this is due to an *in situ* isomerization of the C2 stereocenter at the chromene ring of **GBA**. Literature reports suggest that 2,2-dialkyl chromenes can undergo isomerization under photochemical or thermal conditions, (Kolc and Becker, 1967; Kolc and Becker, 1970; Migani et al., 2005; Garcia et al., 2009; Agua et al., 2021; Jeon et al., 2022), but this process has not been documented in **GBA** or similar **CGXs**. Interestingly, the C6 phenol of **GBA** resonates at 12.75 ppm in CDCl_3_ while that of ***epi*-GBA** resonates at 12.76 ppm. With this in mind, we followed the isomerization of **GBA** (94% d.e.) to ***epi*-GBA** in CDCl_3_ *via* ^1^H NMR spectroscopy (Figure 3A). Heating the solution at 100°C (sealed NMR tube) led to gradual increase in the concentration of ***epi*-GBA** reaching a 72:28 ratio of **GBA:*epi*-GBA** over 4 h. However, no isomerization was observed at 25°C over a period 14 days. We also measured the isomerization of **GBA** in DMSO-*d*6 (Supplementary Figure S8, ESI) and pyridine-*d*5 (Supplementary Figure S9, ESI). After 4 h of heating (100°C), the ratio of **GBA:*epi*-GBA** reached 58:42 and 61:39 in DMSO-*d*6 and pyridine-*d*5, respectively (Figure 3B). Ultimately, a similar ratio (60:40) was observed when heating 1 g of **GBA** in CHCl_3_ at 100°C after 48 h which remained unchanged over a period of 14 days (Supplementary Figure S10, ESI). This reaction was performed in a sealed high pressure reaction vessel that was immersed in an oil bath at 100°C. The results suggest that solvent polarity accelerates isomerization but does not significantly affect the final **GBA:*epi*-GBA** ratio. On the other hand, exposure of **GBA** to visible light using a sunlamp for 24 h at 25°C did not result in any measurable isomerization.

To test whether the carboxylic acid of **GBA** facilitates isomerization, presumably by protonating the chromene oxygen, we synthesized gambogic acid methyl ester **1** (Figure 3C) and followed its conversion to ***epi*-1** *via* ^1^H NMR spectroscopy. We found that there was no significant difference in the rates of C2 isomerization between **GBA** and gambogic acid methyl ester **1** (Figure 3D). However, additional methylation of the C6 phenol led to compound **2**, which was found to undergo epimerization at a considerably slower rate. Specifically, the ratio of **2:*epi*-2** after 4 h of heating at 100°C was measured as 89:11, a remarkable difference compared to **GBA** and **1** (Figure 3D). The combined results suggest that isomerization of **GBA** to ***epi*-GBA** proceeds *via* an *ortho*-quinone methide intermediate (Figure 3E). (Parker and Mindt, 2001; Favaro et al., 2003; Kurdyumov et al., 2003; Majumdar et al., 2012; Day et al., 2017; Song et al., 2017; Roche, 2021) Rupture of the C2-O bond results in an sp (Maneenoon et al., 2015)-hybridized C2 center that can subsequently undergo cyclization from either side of the double bond, resulting in the formation of both **GBA** and ***epi*-GBA** in a 6:4 ratio. The observed erosion of stereochemistry at the C2 center is accelerated under polar solvents and elevated temperatures.

### 2.3 Cytotoxicity profile of GBA

**FIGURE 4:**
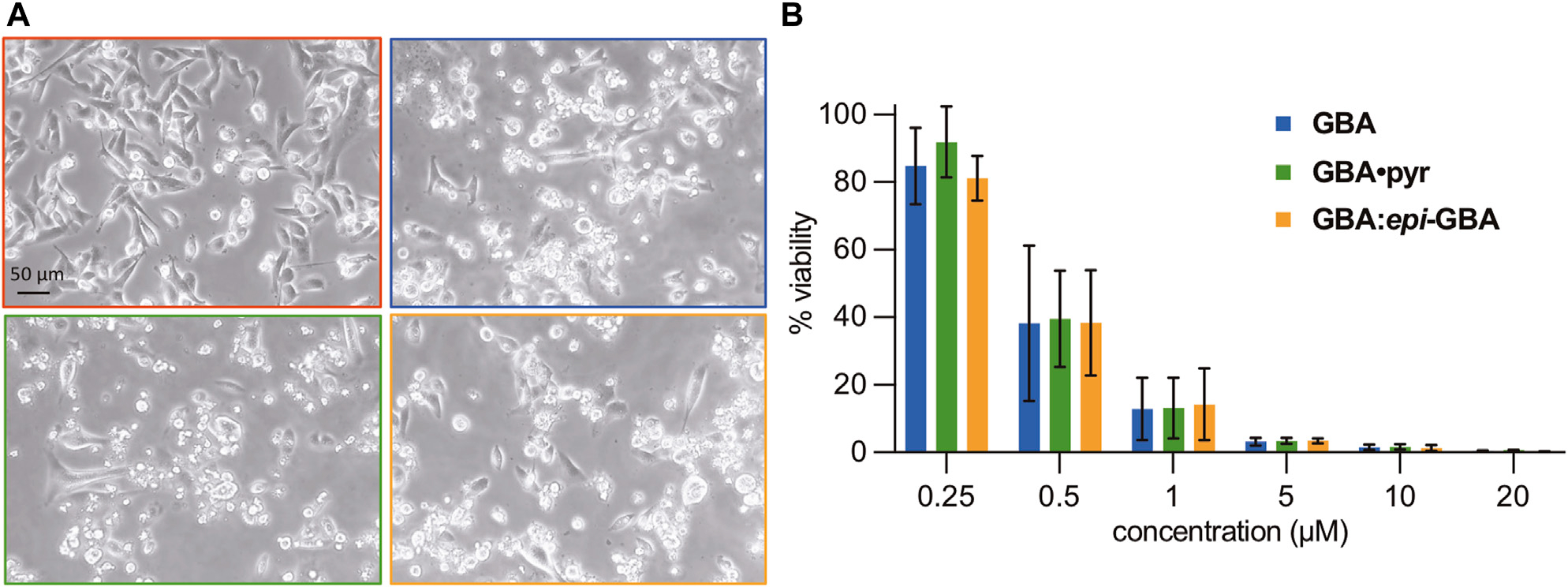
**(A)** Morphological changes in MDA-MB-231 TNBC cells after gambogic acid treatment. Cells were treated for 24 h with DMSO (red panel), or 1 μM each of **GBA** (blue panel), **GBA•pyr** (green panel), **GBA**:***epi*-GBA** (orange panel). In all treatments, except for the DMSO, the cells are rounded and present signs of cell death. Pictures were taken with a Motic AE31 Inverted Microscope using the Motic Image Plus 3.0 software. **(B)** MDA-MB-231 cell viability (%) after gambogic acid treatment. Cells were treated for 24 h with increasing concentrations of **GBA** formulations. Viability (%) over DMSO was calculated using an ATP assay. ANOVA two-factor with replication showed no statistically significant difference between all three formulations.

To evaluate whether the stereochemical erosion at the C2 stereocenter has an impact on the biological profile of gambogic acid, we compared the cytotoxicity of **GBA** (94% de), its pyridinium salt (**GBA•pyr**, 94% de), and an equilibrated mixture of **GBA: *epi*-GBA** (60:40 ratio) against the MDA-MB-231 triple negative breast cancer (TNBC) cell line (Bianchini et al., 2016; Yin et al., 2020; Bharaj et al., 2021). This is a highly metastatic breast adenocarcinoma line characterized by a lack of estrogen, progesterone, and HER2 receptors and thus, it is resistant to all anti-hormonal and HER2 targeted therapeutics. Cells were seeded in 6-well plates and allowed to grow to 70% confluency before treatment with 1 μM of the three formulations of gambogic acid for 24 h (Figure 4A). Using light microscopy, we observed increased numbers of rounded and detached cells in all wells treated with the **GBA** formulations in comparison to the DMSO treated wells (vehicle control). Under the conditions tested, the three **GBA** formulations had similar phenotypic effects on the MDA-MB-231 cells, indicating cell death. Visualization of morphological hallmarks of apoptosis, such as cell shrinkage, membrane blebbing, and apoptotic bodies, led us to hypothesize that apoptosis was the main cell death mechanism (Syed Abdul Rahman et al., 2013). For a more detailed comparison of the cytotoxicity, we performed a luminometric ATP assay to measure viability of the TNBC cell line after 24 h of treatment with different concentrations of the three formulations (Figure 4B). We found that the percent viability of the MDA-MB-231 cells steadily decreased in a dose-dependent manner dropping below 50% at concentrations less than 1 μM. Statistical analysis using ANOVA single factor revealed no significant differences between the three **GBA** formulations for each concentration. It is worth noting that similar submicromolar IC_50_ values have been reported for the triple negative 3D breast cancer spheroids^*MARY-X*^ (Theodoraki et al., 2015). More recently, using the CCK-8 viability assay, the IC_50_ of **GBA** was calculated at 0.4 μg/ml (or 0.64 μM) against the mouse triple negative breast cancer cell line 4T1 and less than 1.0 μg/ml (or less than 1.59 μM) against the MDA-MB-231 cell line (Dang et al., 2021). Overall, our findings corroborate previous studies on **GBA** and related **CGXs** indicating a favorable therapeutic index as evidenced by a selective submicromolar cytotoxicity against all breast cancer subtypes with no apparent detrimental effects in normal human breast epithelial cells (Li et al., 2012; Bai et al., 2015; Chantarasriwong et al., 2019; Triyasa et al., 2022).

**FIGURE 5:**
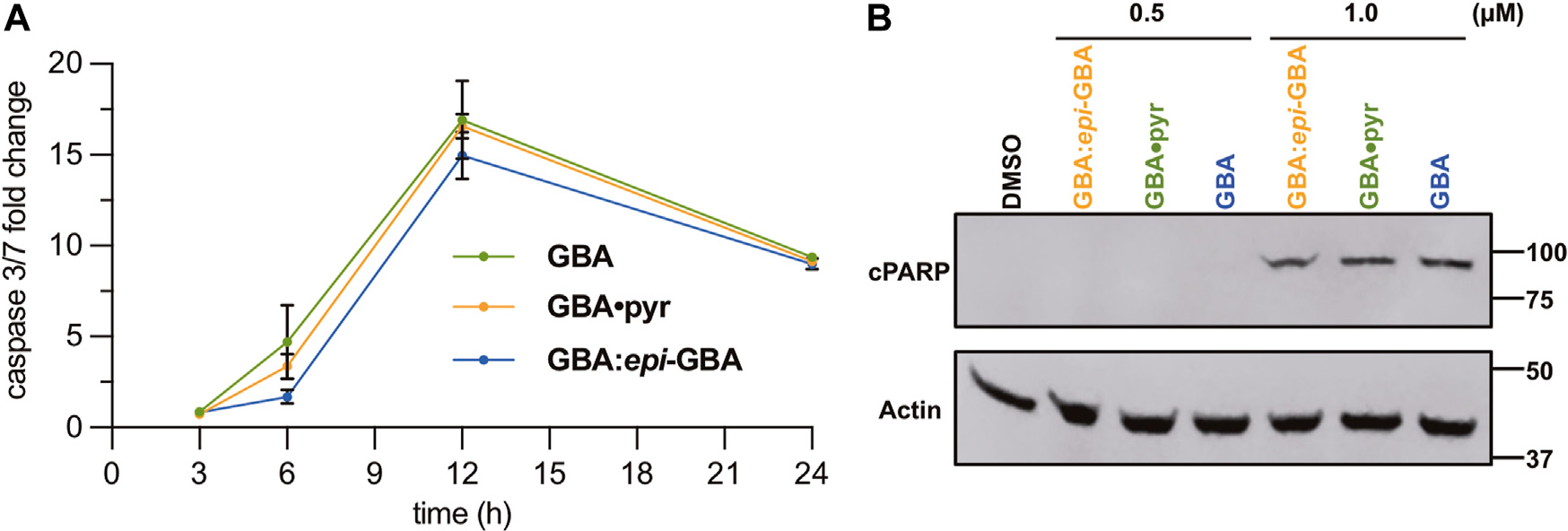
**(A)** Time course analysis of caspases 3/7 activity in MBA-MB-231 cells upon incubation with various GBA formulations (1 μM). Data presented are mean ± SD (*n* = 3). **(B)** Detection of cleaved PARP (cPARP) *via* western blot analysis. MDA-MB-231 cells treated for 24 h with 0.5 and 1.0 μM of **GBA** formulations were lysed and 20 μg of total protein was used for gel electrophoresis. After transfer, the membrane was probed with anti-cPARP, while actin was used as a loading control.

Given the effect of the gambogic acid formulations to the morphology and viability of the MDA-MB-231 cell line (Figures 4A,B), we further investigated the kinetics of induction of apoptosis. To that end, activation of the executioner caspases 3/7 was studied after 3, 6, 12, and 24 h of treatment with 1 μM of each compound (Figure 5A). All three formulations led to similar increase in the activity of executioner caspases 3/7 with the maximum activity reached after 12 h of exposure to 1 μM of the compounds, indicating similar performance in induction of cell death *via* apoptosis. Activation of the executioner caspases leads to proteolytic cleavage of effector proteins and degradation of cellular components (McIlwain et al., 2013). One of the well-known substrates targeted by activated caspases is poly (ADP-ribose) polymerase-1 (PARP-1). Its proteolytic processing by caspases 3/7 generates a catalytic fragment with 89 kDa size (cPARP) that is considered a molecular hallmark of apoptosis (Soldani and Scovassi, 2002; Wong, 2011). With this in mind, we sought to verify the induction of the apoptotic pathway by western blot detection of cleaved PARP (Figure 5B). Treatment of the MDA-MB-231 breast cancer cells with 1 μM of the equilibrated mixture, **GBA•pyr**, or >97% **GBA** epimer, for 24 h was sufficient to allow detection of the cleaved large fragment (89 kDa) of human PARP asserting apoptotic cell death. Similar results were obtained in a colorectal cancer cell model (Wen et al., 2015).

## 3 Conclusion

We report here an efficient multigram scale isolation of gambogic acid (**GBA**) from various sources of commercially available gamboge. The isolation process is based on three steps that include separation of **GBA** and its C2 epimer ***epi*-GBA** from non-organic components, separation of **GBA** from ***epi*-GBA** *via* repeated crystallization as the pyridinium salt and acid-induced liberation of free **GBA**. The overall process reproducibly yields approximately 13 g of **GBA** in >97% diastereomeric purity starting from 100 g of gamboge resin. The usual contaminant of **GBA** is ***epi*-GBA**, a diastereomer that is produced *via* a heat-induced erosion of the C2 stereochemistry. The mechanism of this epimerization is proposed to proceed *via* an *ortho*-quinone methide intermediate that, under equilibration conditions, results in a 6/4 ratio of **GBA:*epi*-GBA**. Biological studies revealed that either pure **GBA**, its pyridinium salt or the equilibrated mixture of **GBA:*epi*-GBA** induce similar levels of cytotoxicity in MBA-MB-231 cells with IC_50_ values at submicromolar concentration and induce apoptotic death as evidenced by activation of caspase 3/7 and cPARP cleavage after 12 h of incubation. Based on the above, it can be concluded that the observed stereochemical erosion accounts for the chemical fragility of **GBA** but does not appear to affect its biological activity at least as relates to its cancer cell cytotoxicity. In turn, the results further support the pharmacological significance of gambogic acid and, together with the multigram-scale isolation, pave the way for a more thorough evaluation of its potential in anticancer drug design and development.

## 4 Experimental section

### 4.1 General information for chemical purification and compounds characterization.

Reactions were monitored by thin-layer chromatography (TLC) carried out on 0.25 mm E. Merck silica gel plates (60F-254) and visualized under UV light and/or by treatment with a solution of CAM or KMnO_4_ stain followed by heating. Flash column chromatography was performed on silica gel (Merck Kieselgel 60, 230–400 mesh) using hexane/ethyl acetate or hexane/ethyl ether as standard eluents. ^1^H NMR and ^13^C NMR spectra were recorded on a 400 or 500 MHz Varian or a 500 JEOL instrument. Chemical shifts δ) are quoted in parts per million (ppm) referenced to the appropriate residual undeuterated solvent peak, with the abbreviations s, bs, d, t, q, dd, m, denoting singlet, broad singlet, doublet, triplet, quartet, doublet of doublets, multiplet, respectively. J is a coupling constant given in Hertz (Hz). High resolution mass spectra (HRMS) were recorded on a VG7070HS mass spectrometer under chemical ionization (CI) conditions, on a VG ZAB-ZSE mass spectrometer under fast atom bombardment (FAB) conditions, or on a Bruker microTOF mass spectrometer under electrospray ionization (ESI) conditions. Specific information on the synthetic/analytical protocols as well as copies of the spectroscopic data for all compounds are shown in the Supporting Information.

### 4.2 Commercial sources of gamboge resin

Commercially available gamboge was purchased from Kremer Pigments (https://www.kremer-pigmente.com/Lot No: 37050), Metropolitan Music (https://www.metmusic.com/, Lot No: 27320) and Wood Finishing Enterprises (https://woodfinishingenterprises.com/, Lot No: 14–1720).

### 4.3 Isolation of GBA from gamboge

Gamboge resin (100 g) was stirred with MeOH (300 ml) for 10 min and then filtered under reduced pressure. The solid residue was further washed with MeOH (2 × 100 ml) and the filtrate was concentrated (under reduced pressure at room temperature) to yield 70–73 g of crude **GBA** as an amorphous orange solid. Spectroscopic evaluation of this material (^1^H NMR) showed that it is composed of ~30% **GBA**, ~25% ***epi*-GBA**, and 45% other non-identified compounds. The crude **GBA** (70 g) was then dissolved in a mixture of pyridine/water: 85/15 (125 ml) and heated at 60°C until completely dissolved (about 10 min). Orange crystals were observed after cooling and the mixture was left for 16 h at room temperature. The resulting crystals (22.9 g) were filtered and dried. Spectroscopic evaluation (^1^H NMR) showed that the resulting material was composed of 76% pyridinium salt of gambogic acid (**GBA•pyr**), 18% of pyridinium salt of epi-gambogic acid (**epi-GBA• pyr**), and 6% of a non-identified compound. This compound was subjected to a second recrystallization with pyridine/water: 85/15 (63 ml) at 60°C to produce 15.8 g of orange crystals composed of 89% **GBA•pyr**, 9% of ***epi*-GBA• pyr** and 2% of a non-identified compound. A third recrystallization with pyridine/water: 85/15 (63 ml) under identical conditions afforded 14.9 gr of orange crystals composed of 97% **GBA•pyr** and 3% of ***epi*-GBA• pyr**. This material was dissolved in diethyl ether (200 ml) and extracted with 15% aqueous hydrochloric acid (200 ml). The ether layer was concentrated to produce an orange solid (13.3 g) composed of 97% gambogic acid (**GBA**) and 3% of epi-gambogic acid (***epi*-GBA**).

### 4.4 Isomerization studies of GBA to *epi*-GBA

GBA (10 mg) were dissolved in CDCl_3_ (0.6 ml) and the resulting orange solution was placed in an NMR tube. The tube was sealed and placed in a heating bath at respective temperatures (80, 100, 120, and 150°C). ^1^H NMR spectra were recorded at predetermined times.

### 4.5 Synthesis of gambogic acid methyl ester (1)

To a 20 ml scintillation vial equipped with a magnetic stirbar was added gambogic acid pyridinium salt (100 mg, 0.14 mmol, 1eq.), acetone (3 ml), potassium carbonate (78 mg, 0.57 mmol, 4 eq.), and methyl iodide (176 μl, 2.83 mmol, 20 eq.). The reaction was allowed to stir at room temperature until complete as determined by TLC (product *R*_*f*_ = 0.6, 40% EtOAc in hexanes). Next, the mixture was diluted with EtOAc and washed with water (5 ml, x2). The organic phase was partitioned, dried over magnesium sulfate, filtered, and concentrated under reduced pressure. The resulting crude mixture was then purified by silica gel column chromatography using a gradient eluent of 30%–40% EtOAc in hexanes to yield the desired product as an orange, amorphous solid (60 mg, 67% yield). ^1^H NMR (500 MHz, CDCl_3_) δ 12.85 (s, 1H), 7.54 (d, *J* = 6.9 Hz, 1H), 6.68 (d, *J* = 10.1 Hz, 1H), 5.94 (td, *J* = 7.4, 1.4 Hz, 1H), 5.44 (d, *J* = 10.1 Hz, 1H), 5.09–5.00 (m, 2H), 3.48 (dd, *J* = 6.9, 4.5 Hz, 1H), 3.43 (s, 3H), 3.31 (dd, *J* = 14.6, 8.1 Hz, 1H), 3.15 (dd, *J* = 14.6, 5.3 Hz, 1H), 2.99 (qdd, *J* = 16.4, 7.4, 1.4 Hz, 2H), 2.52 (d, *J* = 9.3 Hz, 1H), 2.31 (dd, *J* = 13.4, 4.7 Hz, 1H), 2.06–2.01 (m, 2H), 1.81–1.76 (m, 1H), 1.74 (s, 3H), 1.69 (s, 3H), 1.67 (s, 3H), 1.65 (s, 3H), 1.64 (s, 3H), 1.63–1.59 (m, 1H), 1.55 (s, 3H), 1.44 (s, 3H), 1.44–1.32 (m, 2H), 1.29 (s, 3H). ^13^C NMR (126 MHz, CDCl_3_) δ 203.68, 179.12, 167.44, 161.40, 157.65, 136.17, 135.13, 133.65, 131.99, 131.65, 127.89, 124.58, 123.85, 122.27, 116.00, 107.61, 102.56, 100.54, 90.99, 83.97, 83.81, 81.37, 60.51, 51.21, 49.14, 46.92, 42.15, 31.69, 29.98, 29.22, 28.93, 28.12, 25.78, 25.75, 25.19, 22.83, 22.76, 21.70, 20.96, 18.18, 17.72, 14.30, 14.23. HRMS: Exact mass calculated for [C_39_H_47_O_8_]^+^, 643.3265. Found 643.3261.

### 4.6 Synthesis of compound (2)

To a 20 ml scintillation vial equipped with a magnetic stirbar was added gambogic acid pyridinium salt (300 mg, 0.42 mmol, 1eq.), acetone (4.7 ml), potassium carbonate (293 mg, 2.12 mmol, 5 eq.), and methyl iodide (1.3 ml, 21.2 mmol, 50 eq.). The mixture was allowed to stir for 6 days (product *R*_*f*_ = 0.5, 40% EtOAc in hexanes). The mixture was then filtered and concentrated under reduced pressure. Next, the resulting crude mixture was purified by silica gel column chromatography using a gradient eluent of 30%–40% EtOAc in hexanes to yield the desired product as a yellow, amorphous solid (163 mg, 59% yield). ^1^H NMR (500 MHz, CDCl_3_) δ 7.42 (d, *J* = 6.9 Hz, 1H), 6.65 (d, *J* = 10.1 Hz, 1H), 5.94 (td, *J* = 7.2, 1.6 Hz, 1H), 5.53 (d, *J* = 10.2 Hz, 1H), 5.12–5.06 (m, 1H), 5.06–4.99 (m, 1H), 3.80 (s, 3H), 3.48–3.33 (m, 5H), 3.23 (dd, *J* = 14.6, 5.4 Hz, 1H), 3.02–2.90 (m, 2H), 2.50 (d, *J* = 9.3 Hz, 1H), 2.28 (dd, *J* = 13.4, 4.7 Hz, 1H), 2.01 (q, *J* = 7.9 Hz, 2H), 1.74 (s, 3H), 1.68 (s, 3H), 1.67 (s, 3H), 1.64 (s, 6H), 1.58 (s, 2H), 1.53 (s, 3H), 1.43 (s, 3H), 1.38 (dd, *J* = 13.4, 9.5 Hz, 1H), 1.28 (s, 3H). ^13^C NMR (125 MHz, CDCl_3_) δ 204.22, 174.74, 167.45, 159.77, 158.89, 155.35, 136.36, 135.91, 133.72, 132.02, 131.94, 127.62, 127.32, 123.80, 121.92, 116.73, 112.69, 109.91, 107.44, 90.98, 83.89, 83.70, 80.65, 62.21, 51.12, 49.07, 46.83, 42.13, 30.00, 29.43, 28.99, 27.99, 25.78, 25.75, 25.54, 22.75, 22.21, 20.94, 18.24, 17.70. HRMS: Exact mass calculated for [C_40_H_49_O_8_]^+^, 657.3422. Found 657.3426.

### 4.7 Cell culture and treatment

The MDA-MB-231 cell line was obtained from ATCC (HTB-26) and was cultured in RPMI 1640 media (Gibco 21–870-092) supplemented with 10% heat inactivated FBS (Gibco, 10438–026), 2 mM L-Glutamine (Gibco, 25030–081), and 100 U/mL penicillin, 100 μg/ml streptomycin (Gibco, 15140–122). The cell cultures were maintained at 37°C in a humidified atmosphere with 5% CO_2_ and subcultured using trypsin (Gibco, 25300–054) after reaching 80% confluency. The three formulations of **GBA** (equilibrated **GBA**, **GBA•pyr**, or >97% **GBA**) were dissolved in DMSO to a stock concentration of 10 mM. For microscopy studies, the cells were treated at 70% confluency with 1 μM of each formulation or DMSO for 24 h.

### 4.8 ATP assay

Cell viability was assessed by ATP quantification of metabolically active cells using the CellTiter-Glo 3D kit (Promega, G9682) in multiwell plates according to the manufacturer’s instructions. Briefly, MDA-MB-231 cells at a density of 10^4^/well were seeded in opaque 96-well tissue culture plates and allowed to attach overnight. The next day the three formulations of GBA (equilibrated **GBA**, **GBA•pyr**, or >97% **GBA**) were added to final concentrations of 0.25, 0.5, 1, 5, 10 and 20 μM in quadruplicates. DMSO was used as the solvent control and all treatments were performed for 24 h at 37°C. After 1 h of equilibration at room temperature, CellTiter-Glo 3D reagent at a 1: 1 ratio was added to each well. The plates were mixed on a plate shaker for 10 min and end-point luminescence values were recorded with the FilterMax F-5 multimode microplate reader. Percent viability was calculated as RLU post-treatment * 100%/RLU of DMSO.

### 4.9 Caspase 3/7 activity

Initiation of apoptosis *via* induction of caspase 3/7 activity was measured as above using the Caspase-Glo^®^ 3/7 kit (Promega, G8981). Cells in triplicates were treated with 1 μM of equilibrated **GBA**, **GBA•pyr**, or >97% **GBA** for 0, 3, 6, 12, and 24 h. Wells with only media were used as blanks for background luminescence measurements and wells containing cells treated with only DMSO were used as negative controls. Relative light units (RLU) were recorded 1 h after the addition of the Caspase-Glo 3/7 reagent at a 1:1 ratio and fold change was calculated at each time point using the following equation: Foldchange=(treatmentRLU—blankRLU)/negativecontrolRLU.

### 4.10 Western Blotting

MDA-MB-231 cells treated with DMSO, or each of the three GBA formulations at 0.5 and 1 μM for 24 h were lysed using RIPA buffer (Thermofisher, 89901). Total protein extract was quantified using BCA (Thermofisher, 23225) and 20 μg of protein were loaded and analyzed on a 4–12% Bis-Tris Mini Protein Gel. Following gel electrophoresis, the proteins were transferred to a PVDF membrane using the iBlot2 dry blotting system. Membranes were blocked with 5% dry fat-free milk in TTBS and incubated with primary antibodies overnight at 4°C. Primary antibodies used were: anti-PARP (Cell Signaling Technology, 9546) and anti-actin (BD Biosciences, 612656). Secondary antibodies conjugated to IRDye 680RD or IRDye 800CW from LI-COR Biosciences were used to visualize the proteins of interest on the Odyssey Fc Imager with the Image Studio software.

## Supplementary Material

Supplemental Information

## Data Availability

The original contributions presented in the study are included in the article/Supplementary Material, further inquiries can be directed to the corresponding authors.
