# Supplementary material for: Gambogic acid: Multi-gram scale isolation, stereochemical erosion toward epi-gambogic acid and biological profile
Source: Front Nat Prod. Author manuscript; Available in PMC 2024 Aug 29. (PMC11361287; doi:10.3389/fntpr.2022.1018765)

Gambogic acid: multi-gram scale isolation, stereochemical erosion toward epi-gambogic acid and biological profile

Gary E. Arevalo,[a] Michelle K. Frank,[b] Katelin S. Decker,[b] Maria A. Theodoraki,[b]* Emmanuel A. Theodorakis[a]*

[a.] Department of Chemistry and Biochemistry, University of California San Diego, 9500 Gilman Drive, La Jolla, CA 92093-0358, USA.

[b.] Department of Biology, Arcadia University, 450 S. Easton Road, Glenside, PA, 19038.

Electronic Supporting Information

I.	Table of Contents		1
II.	Table 1 and Figures S1-S10		2-11
	Table S1.	Extraction of gamboge resin with various solvents	2
	Figure S1.	^{1}H NMR spectrum of crude GBA in $CDCl_3$	3
	Figure S2.	Profiling the purification of GBA from gamboge	4
	Figure S3.	^{1}H NMR spectrum of GBA•pyr in $CDCl_3$	5
	Figure S4.	^{13}C NMR spectrum of GBA•pyr in $CDCl_3$	6
	Figure S5.	^{1}H NMR spectrum of pure GBA in $CDCl_3$	7
	Figure S6.	^{13}C NMR spectrum of pure GBA in $CDCl_3$	8
	Figure S7.	Partial ^{1}H NMR spectra of the organic components of various gamboge resins	9
	Figure S8.	^{1}H NMR studies of time-dependent epimerization of GBA to *epi*-GBA in DMSO-*d*6 at 100 °C	10
	Figure S9.	^{1}H NMR studies of time-dependent epimerization of GBA to *epi*-GBA in pyridine-*d*5 at 100 °C	10
	Figure S10.	^{1}H NMR of equilibrated mixture of GBA:*epi*-GBA	11
III.	Copies of NMR/MS Spectra of compounds 1,2		12-17

Table S1. Extraction of gamboge resin with various solvents

Solvent	Amount Solvent Utilized (mL)	Amount Crude GBA Obtained (g)
**Acetone**	300	13.8
**ACN**	500	12.6
**Diethyl Ether**	450	7
**DCM**	500	13.7
**MeOH**	200	18

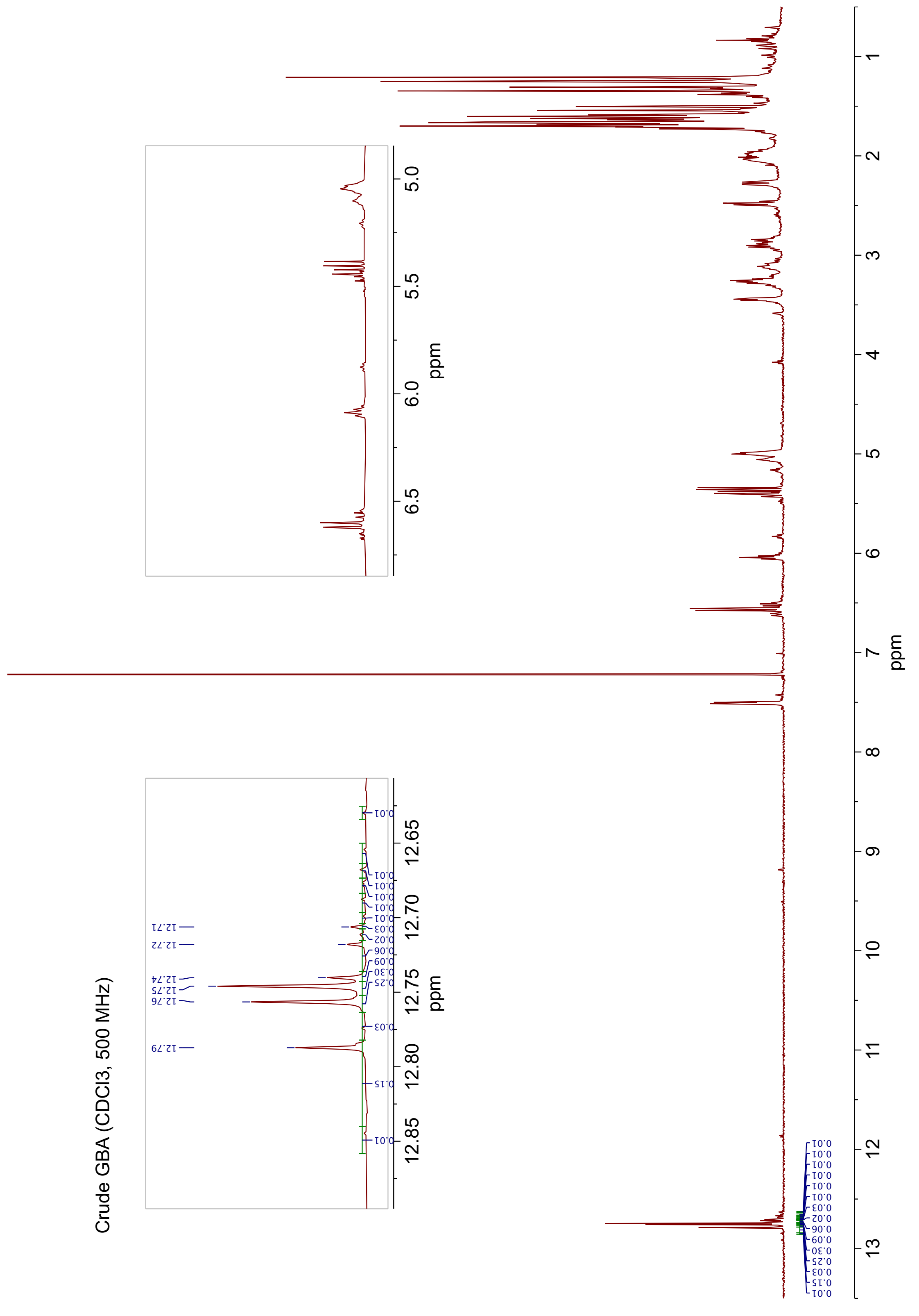

Figure S1. ^{1}H NMR spectrum of crude GBA

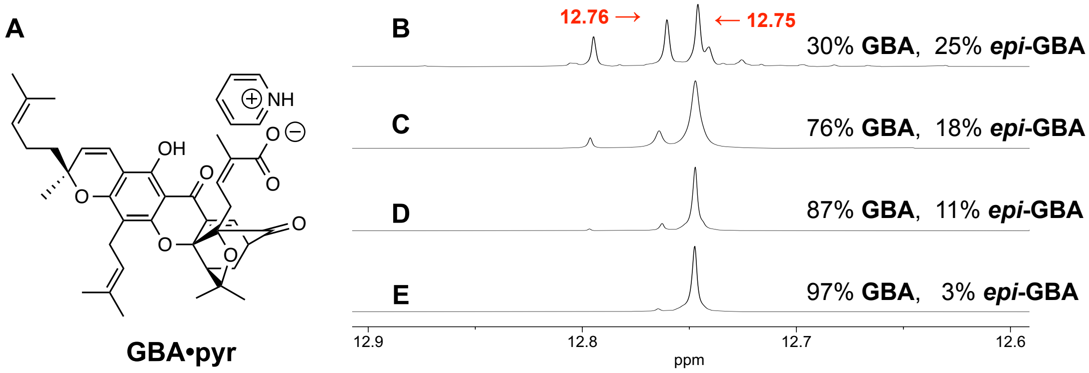

Figure S2. Profiling the purification of GBA from gamboge

(A) Chemical structure of **GBA•pyr**, the pyridinium salt of **GBA**

(B) Partial ^{1}H NMR spectrum showing the representative C6 hydroxyl groups of the various components in crude **GBA**.

(C) Partial ^{1}H NMR spectrum showing the representative C6 hydroxyl groups of the various components in crude **GBA** after one crystallization with pyridine/water.

(D) Partial ^{1}H NMR spectrum showing the representative C6 hydroxyl groups of the various components in crude **GBA** after two crystallizations with pyridine/water.

(E) Partial ^{1}H NMR spectrum showing the representative C6 hydroxyl groups of the various components in crude **GBA** after three crystallizations with pyridine/water.

Note: Spectra C-E are acidified samples of **GBA•pyr**. for ^{1}H NMR analysis.

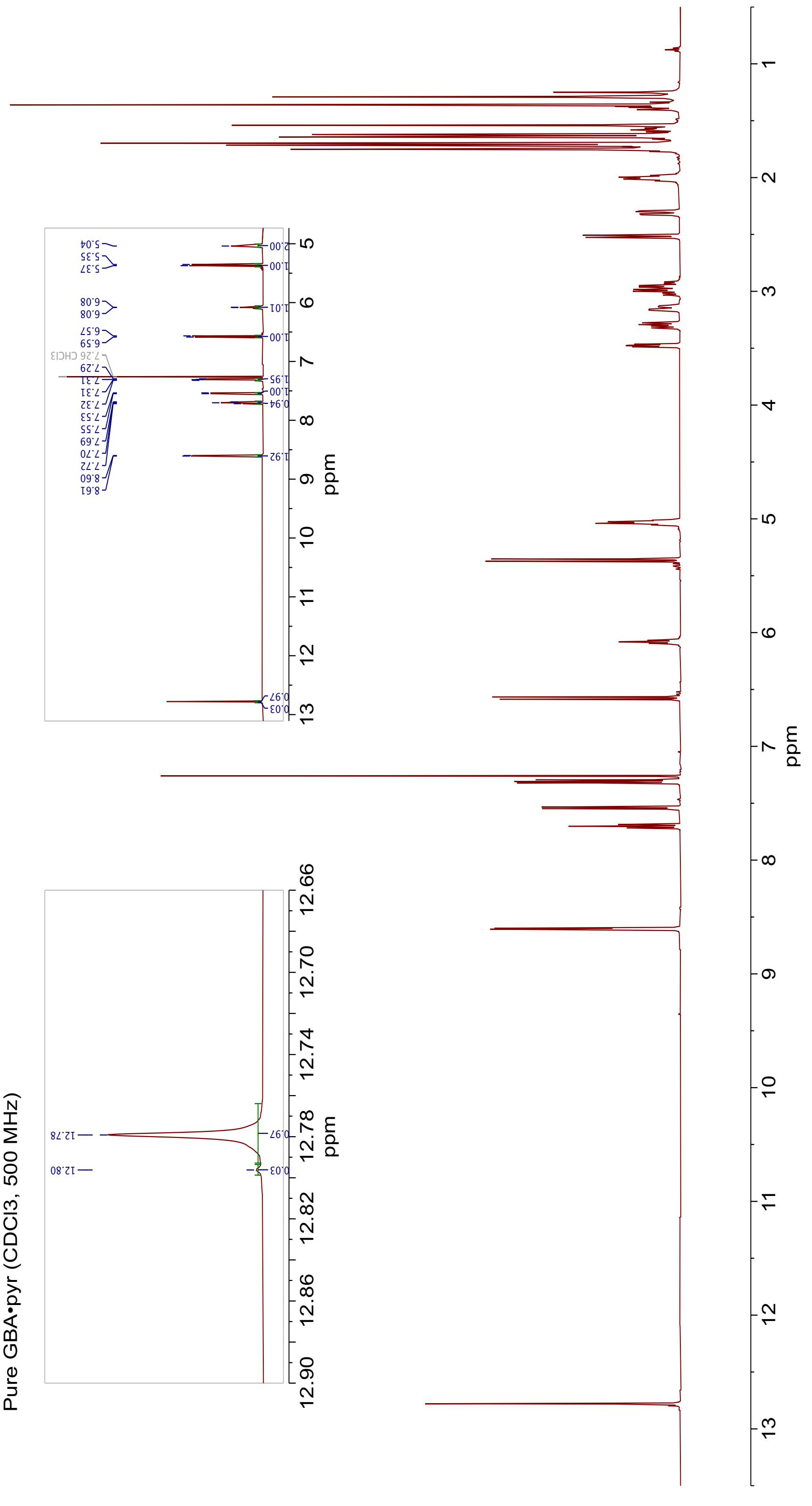

Figure S3. ^{1}H NMR spectrum of GBA•pyr in $CDCl_3$

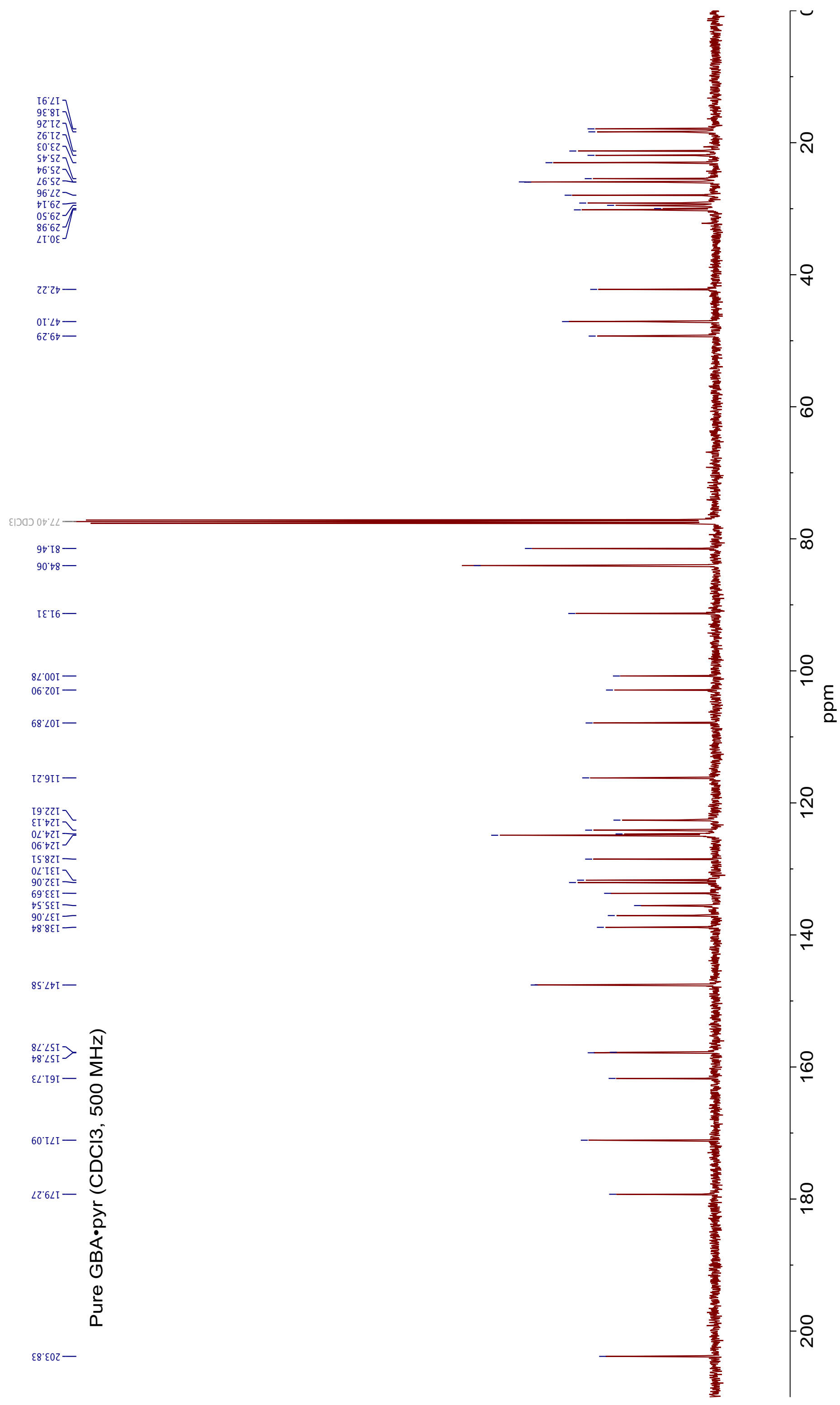

Figure S4. ^{13}**C NMR spectrum of GBA•pyr in** $CDCl_3$

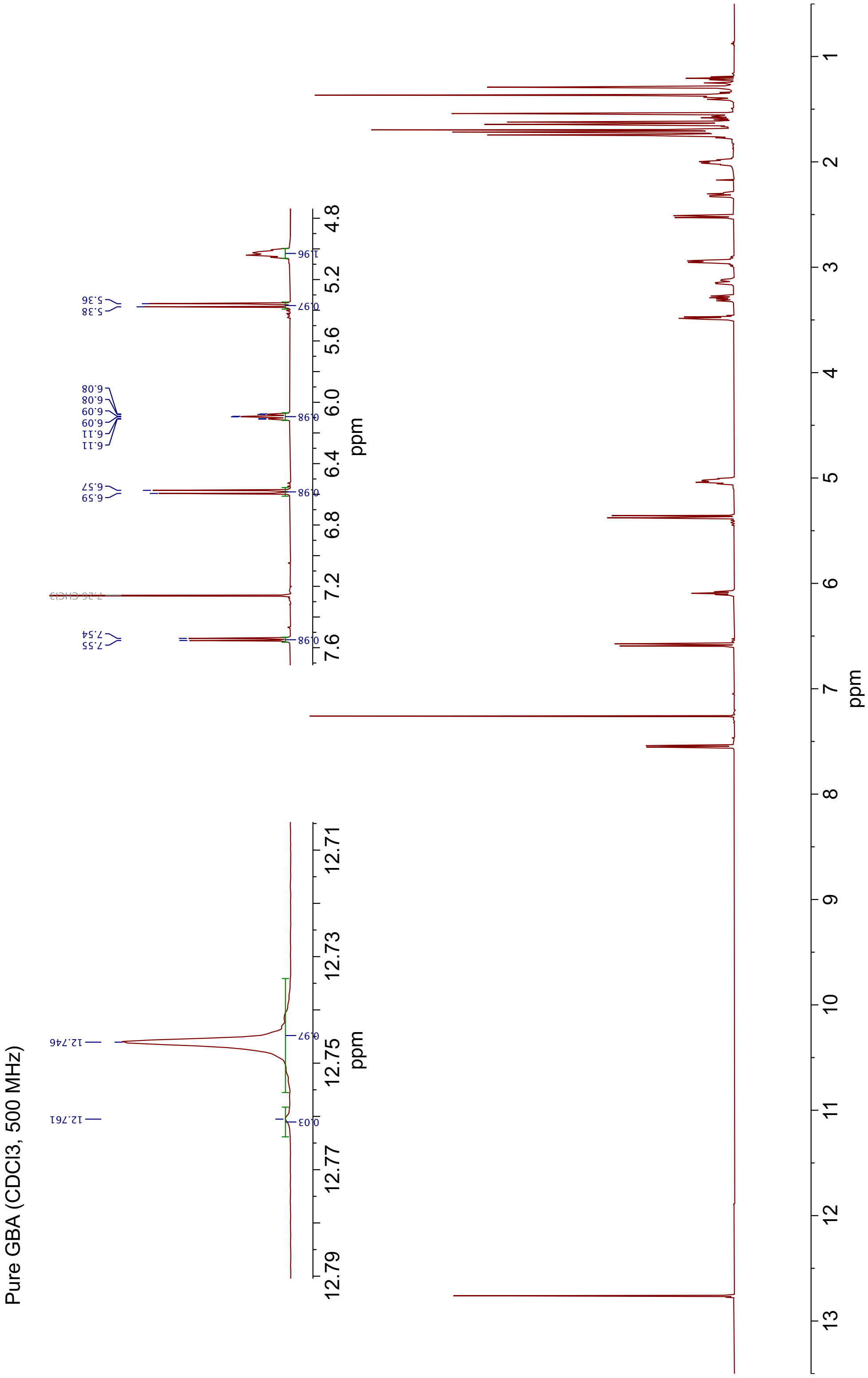

Figure S5. ^{1}H NMR spectrum of pure GBA in $CDCl_3$

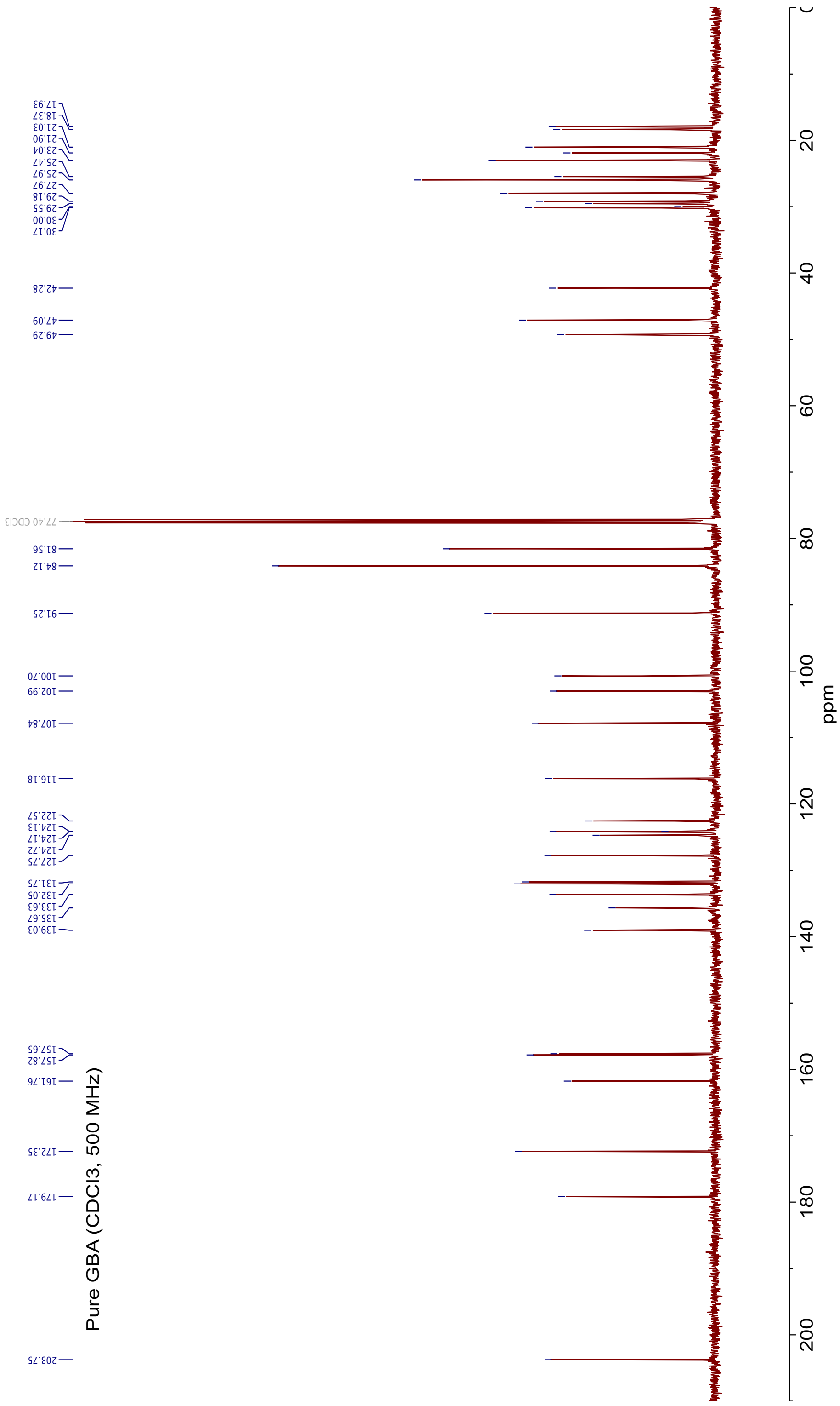

Figure S6. ^{13}C NMR spectrum of pure GBA in $CDCl_3$

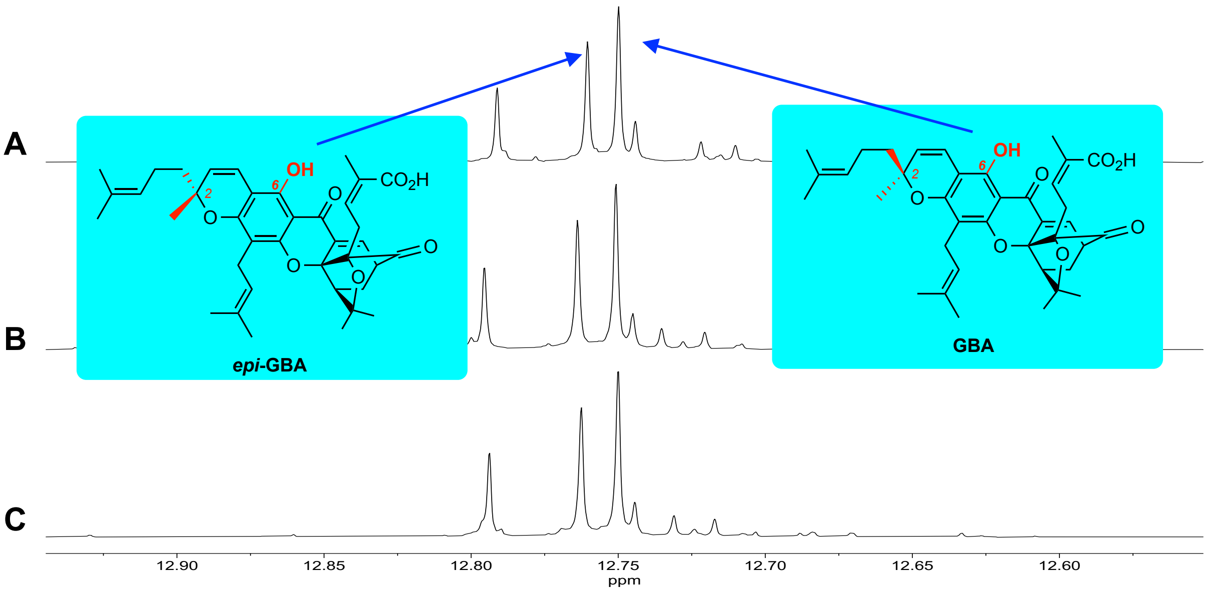

Figure S7. Partial ^{1}H NMR spectra of the organic components of various gamboge resins

(A) Gamboge from Metropolitan Music Company

(B) Gamboge from WoodFinishing Enterprises and

(C) Gamboge from Kremer Pigmente.

Note: The representative C6 hydroxyl group of **GBA** appears at 12.75 ppm and that of ***epi*-GBA** at 12.76 ppm in $CDCl_3$

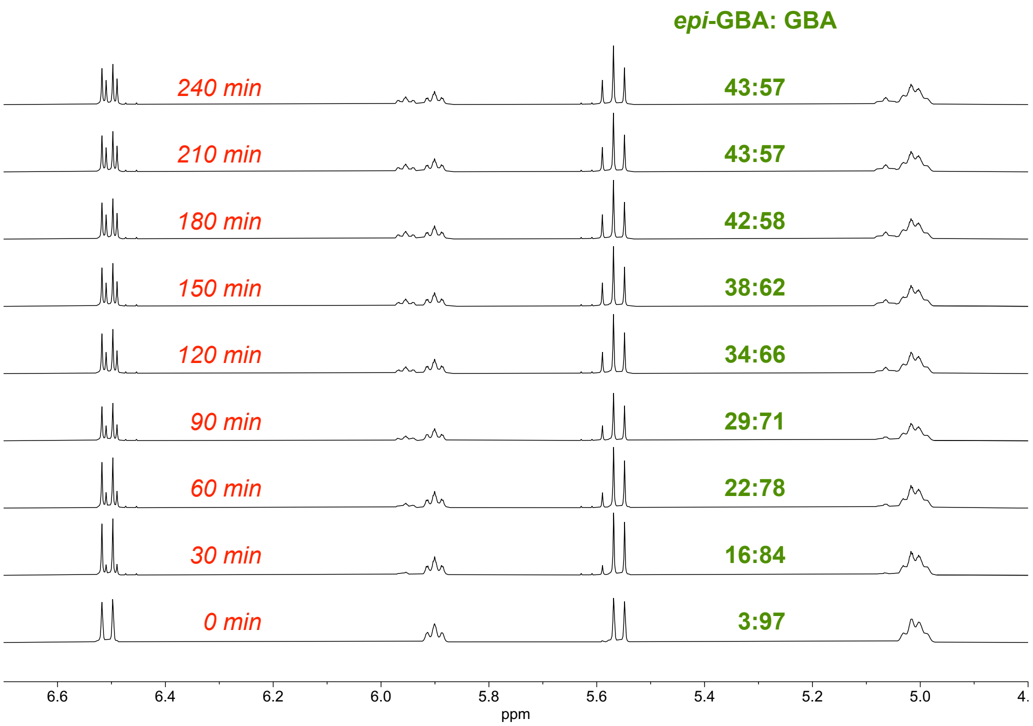

Figure S8. ^{1}**H NMR studies of time-dependent epimerization of GBA to** ***epi*****-GBA in DMSO-*****d*****6 at 100 °C**

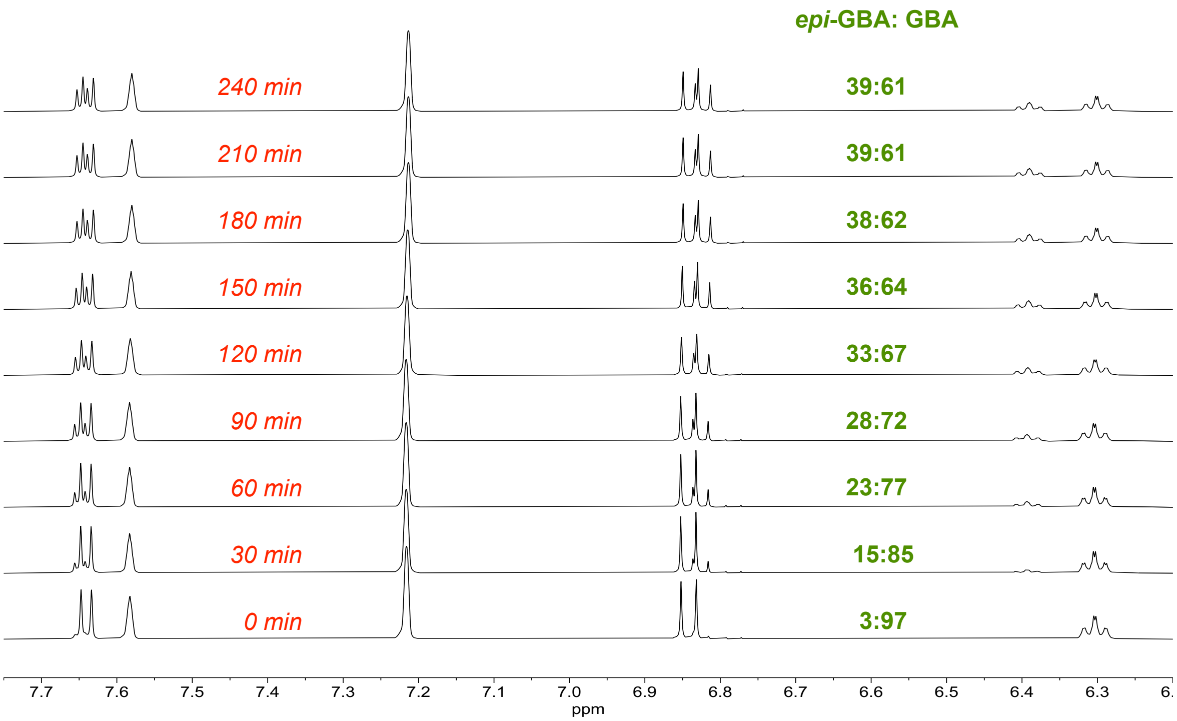

Figure S9. ^{1}**H NMR studies of time-dependent epimerization of GBA to** ***epi*****-GBA in pyridine-*****d*****5 at 100 °C**

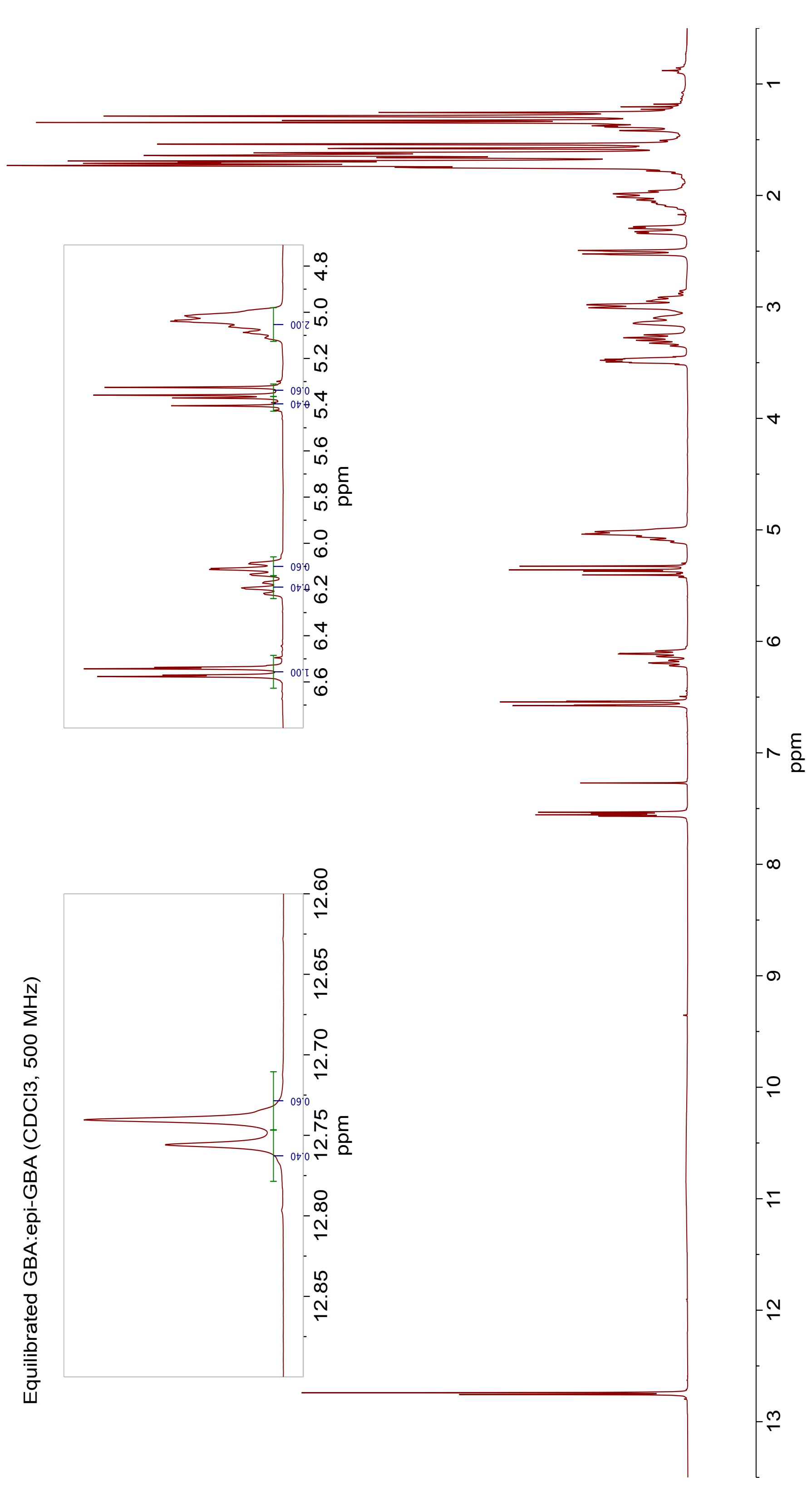

Figure S10. ^{1}H NMR of equilibrated mixture of GBA:*epi*-GBA

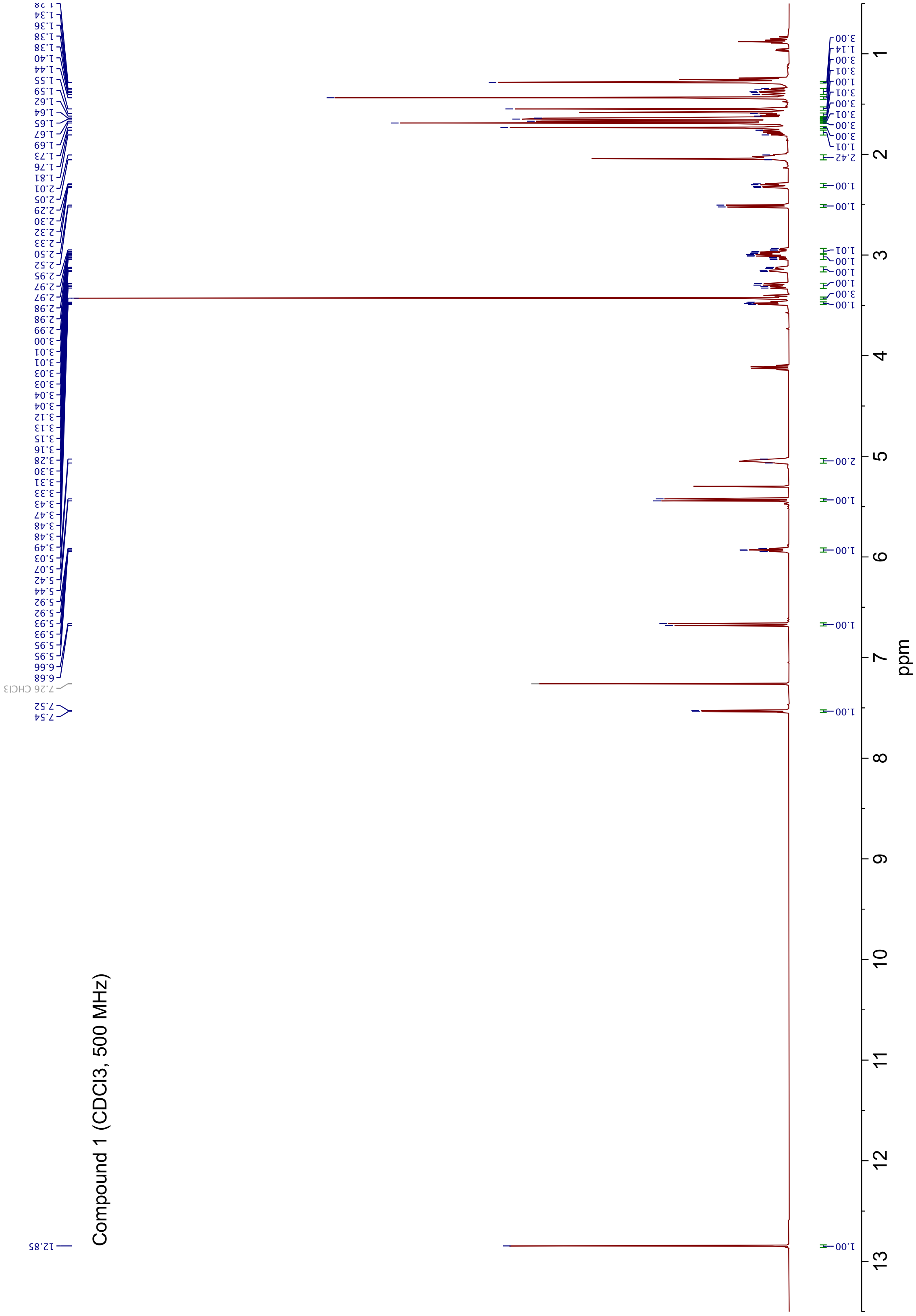

Figure S11. ^{1}H NMR of compound 1

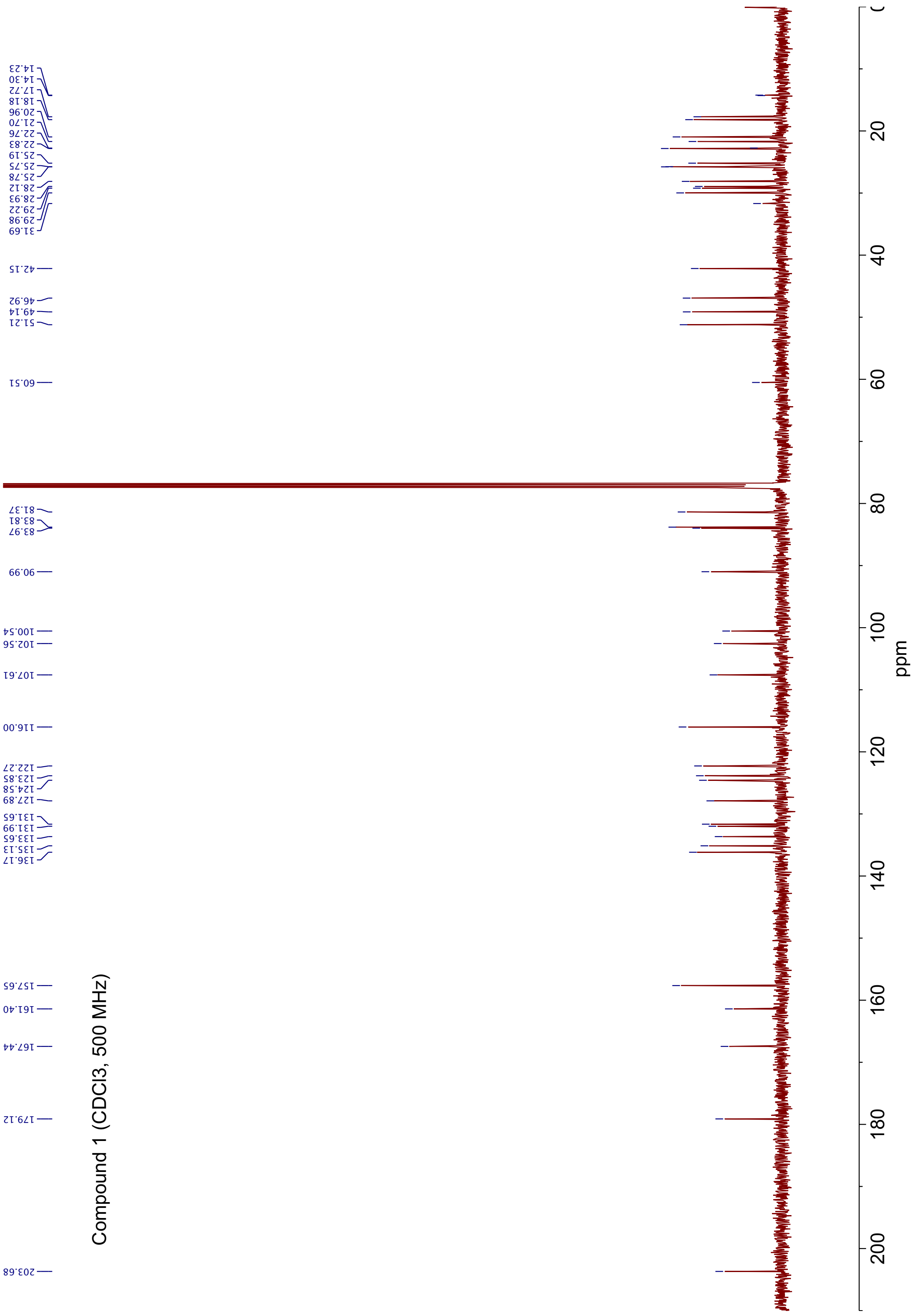

Figure S12. ^{13}C NMR of compound 1

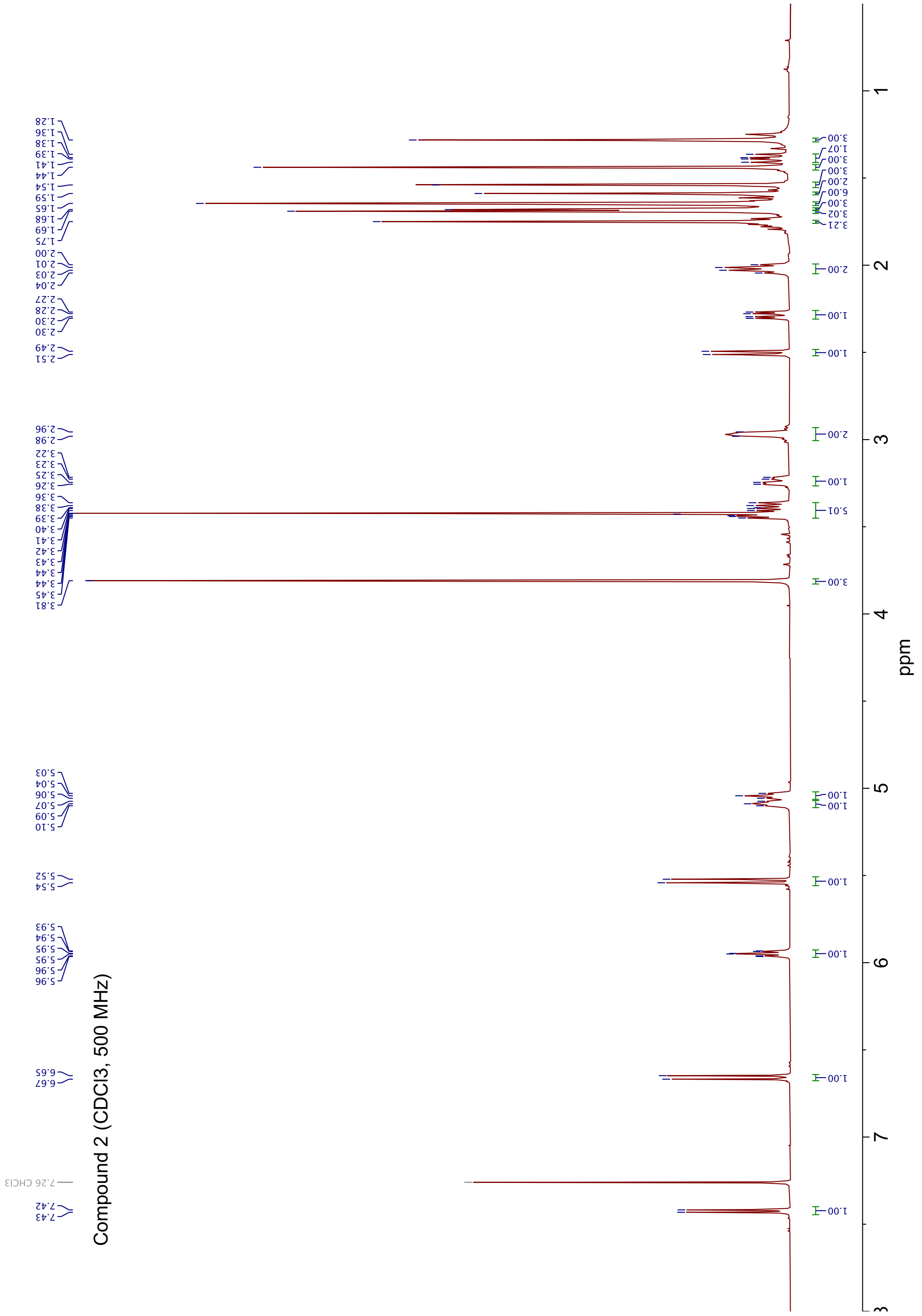

Figure S13. ^{1}**H NMR of compound 2**

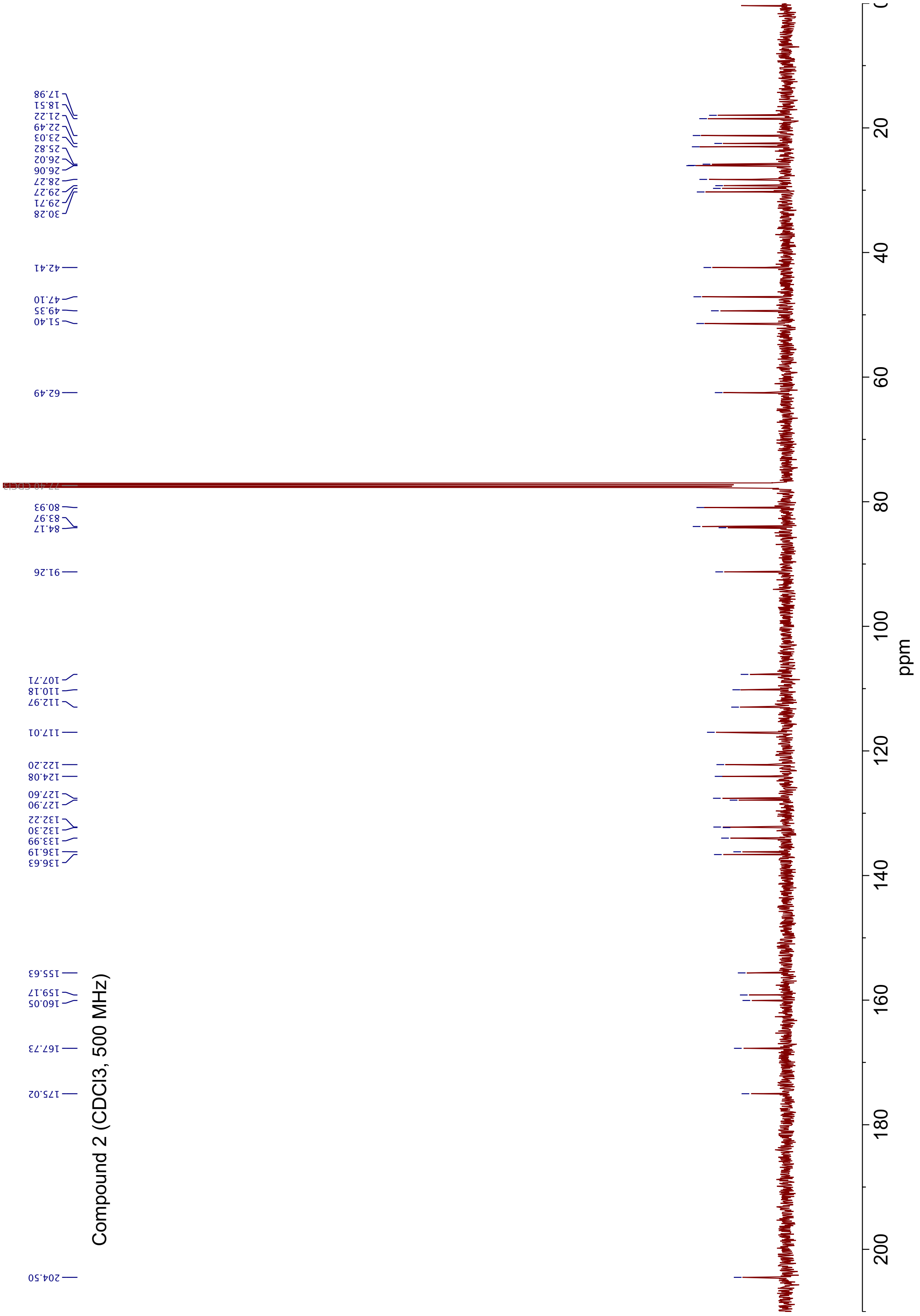

Figure S14. ^{13}C NMR of compound 2

HR-ESI-TOFMS Positive Ion Mode for 1

Mass Measured	Theoretical Mass	Delta (ppm)	Composition
643.3261	643.3265	-0.6	$[C_{39}H_{47}O_8]^+$

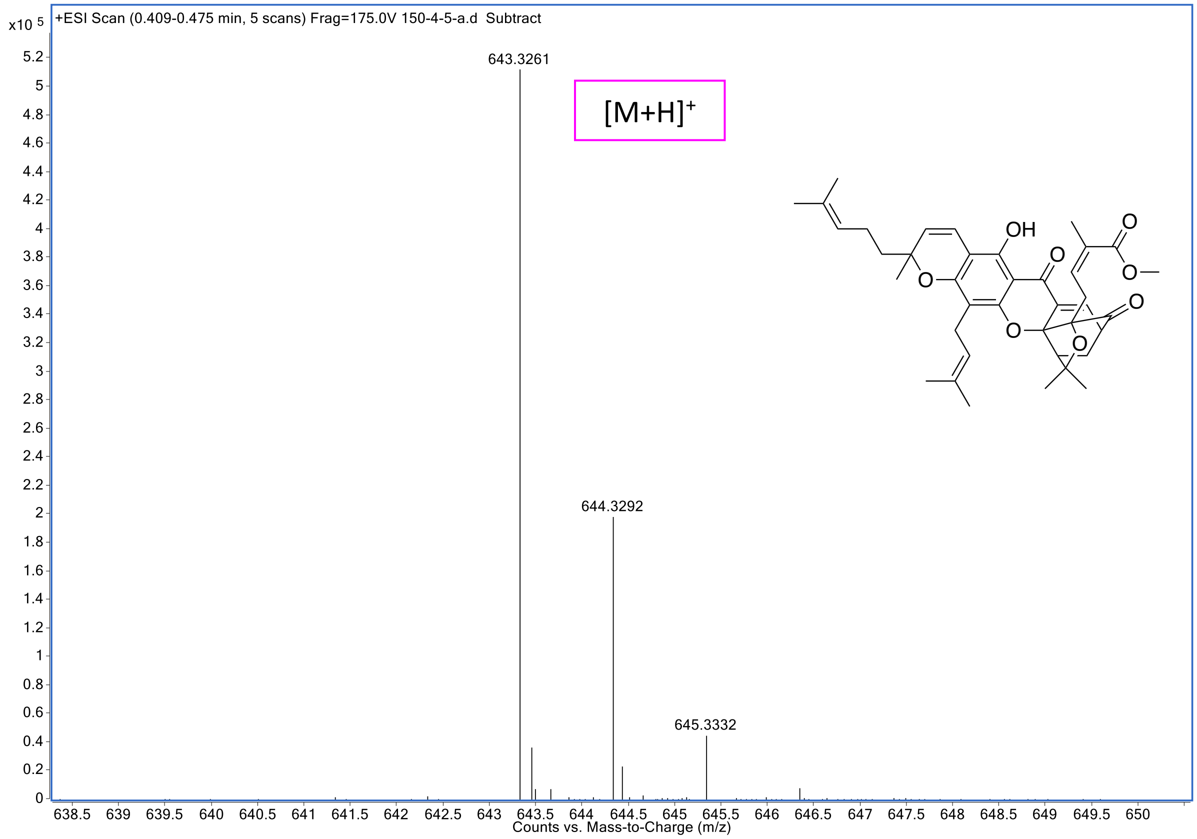

HR-ESI-TOFMS Positive Ion Mode for 2

Mass Measured	Theoretical Mass	Delta (ppm)	Composition
657.3426	657.3422	0.6	$[C_{40}H_{49}O_{8}]^{+}$

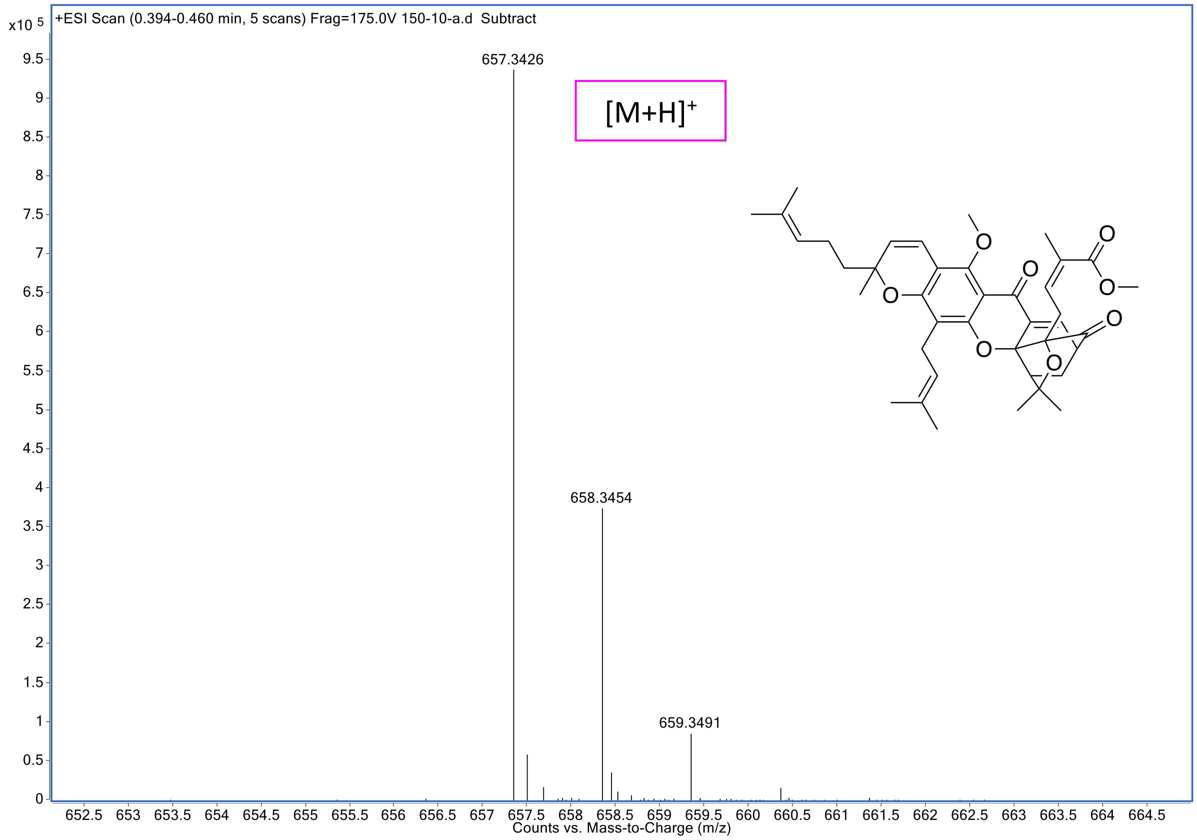

Supplement: Supplemental Information [file NIHMS2017495-supplement-Supplemental_Information.pdf]
